# Disseminating for impact: creating curriculum activities for translational dissemination for the clinical and translational research workforce

**DOI:** 10.3389/fphar.2023.1284662

**Published:** 2023-11-08

**Authors:** Jessica Keim-Malpass, Jennifer Phillips, Karen C. Johnston

**Affiliations:** ^1^ Division of Pediatric Hematology-Oncology, University of Virginia School of Medicine, Charlottesville, VA, United States; ^2^ Integrated Translational Health Research Institute of Virginia (iTHRIV), University of Virginia, Charlottesville, VA, United States; ^3^ Department of Neurology, University of Virginia School of Medicine, Charlottesville, VA, United States

**Keywords:** translational science, clinical research workforce, translational dissemination, dissemination and implementation, dissemination

## Abstract

There has been an increased focus on the practices associated with dissemination for the translation of research to clinical practice and ultimately, policy. Simultaneously, there has been attention placed on the role of the clinical research workforce in supporting optimal dissemination efforts for impact and societal benefit. Curriculums focused on education opportunities for dissemination for translational scientists have been under-reported. The Translational Science Benefits Model (TSBM) is a framework that has been developed to support assessment of clinical and translational research outcomes that measure impact (both in the clinical and community setting) beyond traditional citations in academic journals/bibliometric activities. The TSBM framework outlines more than 30 different facets of impact and can provide a basis for operationalizing broad impacts of research for translational and clinical scientists. Engagement science offers methods and modalities to work with individual stakeholders, and collaborators in a team science model, and engagement with external scholars and society. This article will describe the use of the TSBM framework and engagement science strategies to develop a translational dissemination framework with novel components for evaluation of dissemination and implementation activities. We propose using the translational dissemination framework to guide the development of an educational curriculum for the clinical research workforce. We outline the educational domains and proposed evaluation criteria essential in implementing this innovative translational dissemination educational content for the clinical and translational research workforce.

## Introduction

For nearly 20 years the fields of dissemination and implementation (D&I) have developed within the translational sciences domain to extend basic, clinical, and public health research findings to practice to achieve improved health outcomes for both individuals and populations (Viglione et al., 2023). D&I work seeks to foster eventual clinical implementation of tailored and efficacious interventions in real-world environments, make advancements in public health infrastructure, and translate research findings to inform policy (Shelton et al., 2022). D&I approaches have epistemological underpinnings of pragmatism, supporting the understanding of the essential nature of the underlying complexity of people, communities, and systems in disseminating, adopting, and sustaining interventions within real-world settings and contexts (Mehta et al., 2021; Aves et al., 2017). Previous research has noted that translation can often be slow and inconsistent and that dissemination rarely leads to changes in clinical guidelines or clinical practice alone (Gonzales et al., 2012).

Unlike more specialized scientific or clinical disciplines, D&I activities and research span numerous scientific fields, methodological approaches, and health research settings across the translational spectrum from bench research to society (Norton et al., 2017). When Norton and colleagues (2017) mapped the networks of researchers engaged with D&I activities, they found very active engagement of existing researchers in well-defined and small scientific networks (i.e., very similar author networks within similar disciplinary backgrounds and limited diversity of the researchers). Norton and colleagues’ network analysis pushes us to consider how to re-envision and include emerging translational scientists across disciplinary domains within D&I activities.

D&I sciences have been embraced as critical concepts within the lifecycle of translational researcher (Meissner et al., 2020; Shelton et al., 2022). While few have argued the growing importance of the D&I sciences, there has been less attention placed on how to educate the clinical research workforce (defined broadly as early/middle/senior career scientists, research staff associated with laboratory and clinical research settings, members of the scientific, geographic, or illness communities) to inspire translational research efforts. The Clinical and Translational Science Awards (CTSAs) are funded through the National Institutes of Health/National Center for Advancing Translational Science (NCATS) and include a focus on workforce development of clinical and translational researchers. NCATS defines translation broadly as the process of turning observations into interventions that are adopted and sustained to improve health (Mehta et al., 2021). CTSAs fund translational research infrastructures in over sixty academic medical research centers and enable multidisciplinary investigators to 1) facilitate translational research and training across the translational continuum (e.g., basic, clinical, population sciences); 2) provide training to facilitate workforce development, and 3) develop, demonstrate, and disseminate effective research tools and solutions to overcome translational roadblocks (Shelton et al., 2022).

The end goal of D&I integration is to ultimately improve the quality and impact of translational research to improve the health of individuals and communities (Mahoney et al., 2022). To this end, CTSA hubs offer a prime environment to integrate translational dissemination strategies and education curriculum to reach the basic, clinical, and population health research workforce, early-stage investigators (i.e., the K Scholars) and the general communities the CTSAs work within and serve. Emerging scholars and scholar communities often note a gap in their own scientific backgrounds and training in that they want their impact to stretch beyond traditional academic or scientific communities, yet they are not explicitely taught how to disseminate for impact or approach dissemination from an equity-oriented perspective. The epistemological approaches and methodological decisions that support co-created research designs and dissemination plans with communities of interest are often counter to the traditional clinical and translational scientific methods. Therefore, perspectives that integrate novel approaches to D&I efforts are needed to inspire collective action.

Previous scholars have developed core D&I domains for education and integration, particularly for use within the CTSA context and environment (Leppin et al., 2021; Mehta et al., 2021; Mahoney et al., 2022; Shelton et al., 2022) We propose building on previous work to further develop these educational opportunities through a novel translational dissemination framework to guide the development of an educational curriculum for the clinical research workforce. The translational dissemination framework requires more broadly defining scientific activities that lead to impact in the health of individuals and communities. It also requires purposeful integration of a health equity orientation through the development and evaluation of key activities. We will outline the processes, educational domains, and evaluation criteria essential in implementing the translational dissemination education curriculum for the clinical and translational research workforce.

## The translational science benefits model

The Translational Science Benefit Model (TSBM) was developed in 2018 by interdisciplinary translational scientists at Washington University in St. Louis (Luke et al., 2018) The purpose of TSBM was to broadly define scientific activities that lead to downstream impact in areas of clinical/medical, public health, economic/innovation, and policy/legislative impacts and advances (Luke et al., 2018). The TSBM benefits were identified using Delphi process with the ultimate goal of two phases of translation - the first being more traditional dissemination of research results through manuscripts and conferences for a scientific audience, and the second phase including dissemination to a broader audience which includes clinicians, policymakers, health advocates, communities, and funders (Luke et al., 2018; Takagi-Stewart et al., 2023).

Engagement science has been introduced as a central process representing specific methodologies related to translational sciences and D&I (Meissner et al., 2020). Engagement science is very closely linked to methodologies supported by community-based participatory research (CBPR) and action research in that it include bidirectional communication, collaboration, reciprocity, transparency, and trust (Meissner et al., 2020; Skinner et al., 2015; Weitzman et al., 2018) When these approaches are used in conjunction with the TSBM, the process allows for early and ongoing communication and centering of priorities that allow for dissemination activities to be conceptualized and acted upon much further upstream in the research process.

**FIGURE 1:**
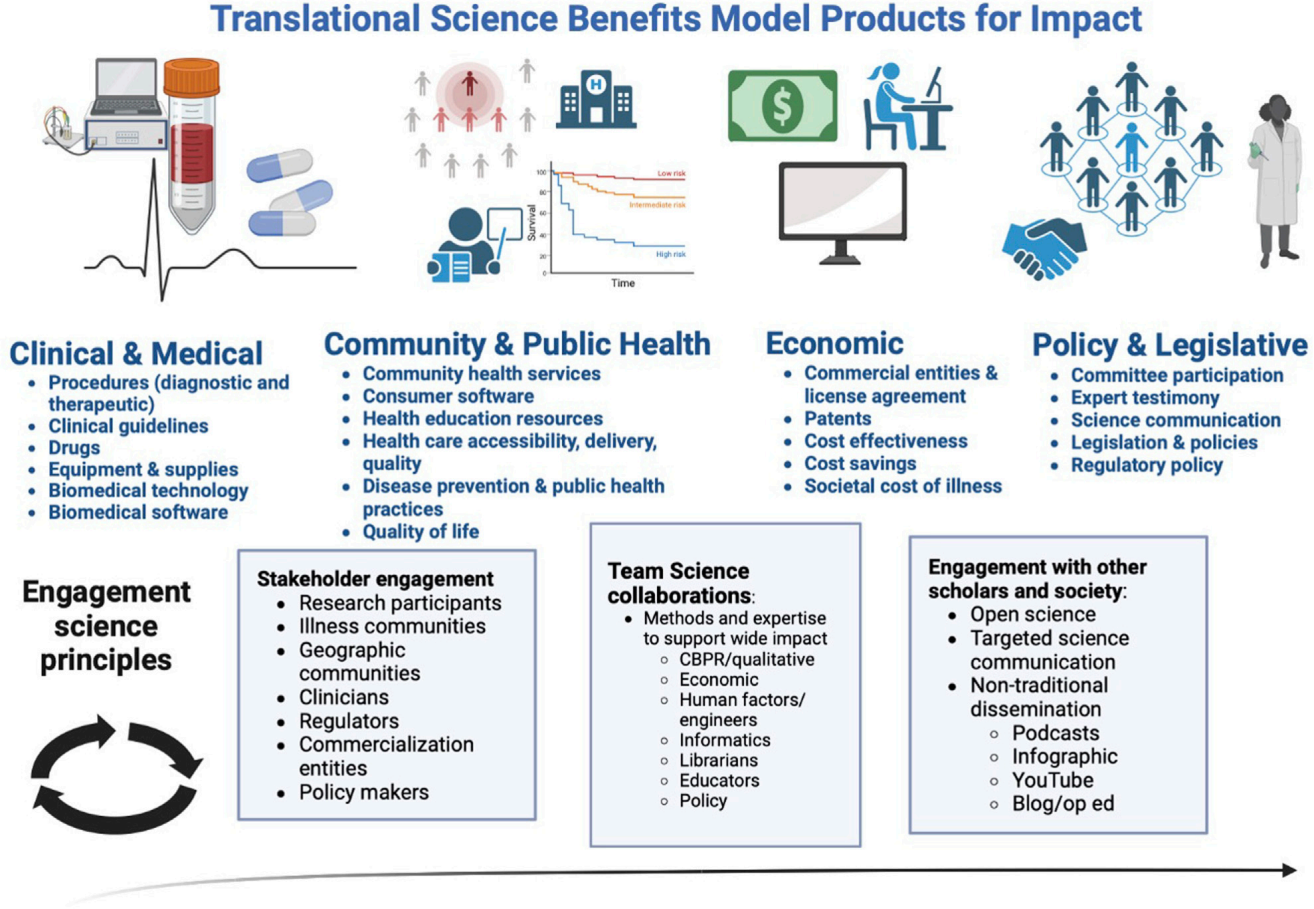
Translational Dissemination Framework: Intersection of Translational Science Benefit Model and engagement science principles across the research lifecycle.

Novel frameworks are needed to support translational dissemination in a manner that is equity-oriented, or working to reduce the power imbalances represented by research participants, illness-oriented communities, historically marginalized groups, and/or geographic communities (Baumann et al., 2023). The clinical and translational science workforce represents individuals from a wide range of prior educational experiences and multidisciplinary background. Core content focused on translational dissemination concepts and techniques is an area of needed attention. Herein, we propose the translational dissemination framework which represents an intersection of the TSBM model, engagement science and equity-oriented principles across the research lifecycle (Figure 1). In this figure, the TSBM broadly defines products for impact across the clinical and medical, community and public health, economic, and policy and legislative sphere. Simultaneously engagement science principles allow for methodological perspectives that allow researchers to actualize products of impact through a patient/community-centered and equity-oriented approach.

## Current curriculum content and plans for the future

We are in the process of expanding our D&I core at the hub integrated Translational Health Research Institute of Virginia (iTHRIV), an NIH-NCATS funded CTSA Hub. Our current educational activities seek to introduce key translational dissemination concepts while also developing an environment to interact with other scientists and research staff interested in D&I engagement (i.e., clinical pharmacists, clinical research coordinators, engineers, data scientists, statisticians, and health disparities researchers all meeting in the same forum to discover their joint interest in equity-oriented approaches to technology-enabled medication adherence). Future work involves extending the curriculum offerings and continually assessing uptake and reach. Table 1 highlights the proposed content delivery examplars and learning environment mapped to the translational dissemination domain. Learning objectives for these curriculum activities include: 1) Identify priority translational dissemination goals and supporting activities for your own program of research or research role; 2) Increase familiarity with engagement science methods and approaches to increase stakeholder engagement throughout the research lifecycle; 3) Identify community partners with diverse experiences and expertise that can be partners in research; 4) Increase networking activities to Identify scientists with complementary skill sets that could collaborate on team science to support translational dissemination.

**TABLE 1:** iTHRIV Planned translational dissemination content exemplars and learning environment.

Domain	Content delivery exemplars	Learning environment
Methods for stakeholder engagement	Community engagement studios	Online focus groups where members of various stakeholder groups are consulted and compensated for their time (CTSA-wide)
Team science collaboration	Team translational science projects	Small group projects where K Scholars use team science approaches to develop and conduct a translational science project (K Scholars)
Engagement with outside scholars and society	Dissemination and implementation consultative service	Drop-in online sessions where any aspect of D&I can be introduced for a topic of discussion; principles of open science are reinforced; overview of non-traditional dissemination; methods and frameworks to support D&I; importance of stakeholder engagement across the translational science research lifecycle (CTSA-wide). Provides context for current gaps in training
Introduction to the Translational Science Benefits models to conceptualize dissemination for impact	Intersection of Dissemination & Implementation CTSA Core and K Scholars program	Recorded online learning videos; framework to guide guest speakers of the K Scholars program (CTSA wide & K Scholars)

These learning activities are intended to be delivered in an online environment with opportunities for real-time engagement with multiple sessions to allow for full concept engagement. The various curriculum activities are meant to take place over the course of a calendar year (August through late July).

## Evaluation

Metrics for uptake, reach, and adoption are central to the ongoing evaluation process. Gonzales and colleagues previously developed competencies for translational researchers engaged in D&I sciences including the following (Gonzales et al., 2012).

• Use theories and methods of multiple disciplines in developing integrated research frameworks *[can be quantified through bibliometric analyses]*
• Integrate concepts and methods from multiple disciplines in designing interdisciplinary research protocols *[can be assessed through collaborative contributions of team members on a research study protocol]*
• Investigate hypotheses through interdisciplinary research [*can be quantified by assessing the educational background and department affiliation of members of the research team]*
• Draft funding proposals/grants for interdisciplinary research programs *[can be quantified by assessing agency and disciplinary breadth of grant funding applications submitted]*
• Disseminate interdisciplinary research results both within and outside the discipline - including both journals and conference presentations *[can be assessed through bibliometric analysis and network analysis of authorship]*
• Author publications with scholars from other disciplines *[can be assessed through bibliometric analysis and network analysis of authorship]*

The D&I evaluation competencies that (Gonzales et al., 2012) proposed can be extended by allowing for a larger scope of translational products that define impact across the research lifecycle such as the impact products included in the TSBM model (Luke et al., 2018). Shea and colleagues also extend D&I domains and competencies by adding elements incorporated through the engagement sciences such as the centering of community engagement and contextual learning within the evaluation components (Shea et al., 2017) These evaluation domains include items such as (exemplars chosen only) (Shea et al., 2017).

• Level of introspection and openness *[can be assessed through self-reflection]*
• Knowledge of stakeholder/community characteristics *[can be assessed through understanding of demographics, historical events, examination of power dynamics through co-created needs assessments]*
• Ability to organize the partnership in a way that facilitates collective decision-making and the ability to adapt to the needs of the community through the research process *[can be assessed through collaborative selection of implementation framework, intervention(s), outcomes, dissemination plans, observation of formal and informal processes of decision making]*
• Assessment of communication effectiveness *[can be assessed through the use of plain language, active listening]*
• Assessment of equitable distribution of resources and credit *[can be assessed through inclusion as authors on manuscripts, grants, provision of equity in resource allocation in budgets]*
• Sustainability of partnership *[can be assessed through history of partnerships, stakeholders/partners become self-sustaining, ongoing time and commitment of effort]*

Baumann et al. (2023) present guiding principles to healthcare equity in D&I science which must also be incorporated in future evaluation components (Baumann et al., 2023).

• Racism must be recognized as a fundamental driver of healthcare inequities *[can be assessed through analysis of written curriculum documentation and video transcripts]*
• Multisector partnerships *[can be assessed through engagement science domains]*
• Active engagement of community members *[can be assessed through engagement science domains]*
• Contextual understanding of healthcare delivery and impact on communities *[can be assessed through engagement science domains]*
We posit extending these evaluation components by including measures associated with:
• Community-engaged results return of research findings (either on an individual or community level) *[can be assessed through frequency and modality of results return]*
• Economic assessments that include distributional cost effectiveness and assessments of equity impacts *[can be assessed through analysis of curriculum documentation and eventual practices]*
• Centering of impact of interventions on patients, families, clinicians, and other end-users *[can be assessed through representation of outcome measures and team science nature of the proposal using methods that focus on end-user experience]*
• Use of open science practices *[can be assessed through bibliometric analysis of available documentation of key research stages, results, manuscript, and study data availability]*
• Use of public-engaged non-traditional dissemination strategies *[can be assessed through quantity of infographics, podcasts, YouTube videos, virtual abstracts]*
• Sustained team science collaboration *[can be assessed through network analysis of multidisciplinary approaches used over time and expansion of team across projects]*

Further engagement with our own stakeholders is needed to co-design and finalize collaborative evaluation frameworks for equity-oriented translational dissemination that include the CTSA D&I, community engagement, and research workforce core groups, as well as the training programs (K and T Scholars). An optimal framework for translational D&I evaluation includes wide ranging products of dissemination incorporated within the TSBM framework, an orientation that centers health equity, along with methodological approaches and contextual learning supported through the engagement sciences. Expanding the core competencies through integration with TSBM products of impact and components of the translational dissemination framework will be the product of future work of our CTSA.

## Conclusion

Translational scholars have thoughtfully outlined the central importance and requirements of D&I components within national CTSA development (Leppin et al., 2021; Mehta et al., 2021; Mahoney et al., 2022; Shelton et al., 2022). At our local NIH-NCATS funded CTSA hub, iTHRIV, we are implementing the dissemination components through the actualization and evaluation of a novel translational dissemination framework that 1) expands products of impact through the TSBM model to include a very broad view of dissemination activities that impact the health and wellbeing of individuals and communities, 2) integrates methodological approaches central to engagement sciences. Over time, we will evaluate the translational dissemination framework for equity-oriented health impact. We anticipate that this framework is one approach that allows translational researchers to actualize the products of impact through patient-and community-centered approaches. We recognize that future engagement with diverse stakeholders in the D&I community is needed to finalize key concepts and approaches. Further, educational model testability needs to be explicated through variable operationalization and measurement of long-term impact.
